# A microfibril assembly assay identifies different mechanisms of dominance underlying Marfan syndrome, stiff skin syndrome and acromelic dysplasias

**DOI:** 10.1093/hmg/ddv181

**Published:** 2015-05-15

**Authors:** Sacha A. Jensen, Sarah Iqbal, Alicja Bulsiewicz, Penny A. Handford

**Affiliations:** Department of Biochemistry, University of Oxford, South Parks Rd, Oxford OX1 3QU, UK

## Abstract

Fibrillin-1 is the major component of the 10–12 nm diameter extracellular matrix microfibrils. The majority of mutations affecting the human fibrillin-1 gene, *FBN1*, result in Marfan syndrome (MFS), a common connective tissue disorder characterised by tall stature, ocular and cardiovascular defects. Recently, stiff skin syndrome (SSS) and a group of syndromes known collectively as the acromelic dysplasias, which typically result in short stature, skin thickening and joint stiffness, have been linked to *FBN1* mutations that affect specific domains of the fibrillin-1 protein. Despite their apparent phenotypic differences, dysregulation of transforming growth factor β (TGFβ) is a common factor in all of these disorders. Using a newly developed assay to track the secretion and incorporation of full-length, GFP-tagged fibrillin-1 into the extracellular matrix, we investigated whether or not there were differences in the secretion and microfibril assembly profiles of fibrillin-1 variants containing substitutions associated with MFS, SSS or the acromelic dysplasias. We show that substitutions in fibrillin-1 domains TB4 and TB5 that cause SSS and the acromelic dysplasias do not prevent fibrillin-1 from being secreted or assembled into microfibrils, whereas MFS-associated substitutions in these domains result in a loss of recombinant protein in the culture medium and no association with microfibrils. These results suggest fundamental differences in the dominant pathogenic mechanisms underlying MFS, SSS and the acromelic dysplasias, which give rise to TGFβ dysregulation associated with these diseases.

## Introduction

Human fibrillin-1 is a 350 kDa glycoprotein that constitutes the major structural component of the 10-12 nm diameter extracellular microfibrils of metazoan species. In addition to conferring structural support to tissues and providing a scaffold for the deposition of elastin during elastogenesis (1–3), fibrillin microfibrils are involved in multiple interactions with cells and other connective tissue components that function to regulate the production of extracellular matrix. These include the binding of cells to fibrillin-1 via the integrins αvβ3, α5β1 and αvβ6 (4–6), and the sequestration on microfibrils of growth factors such as transforming growth factor-β (TGFβ) and the bone morphogenetic proteins (BMPs) (7–9).

**Figure 1.**
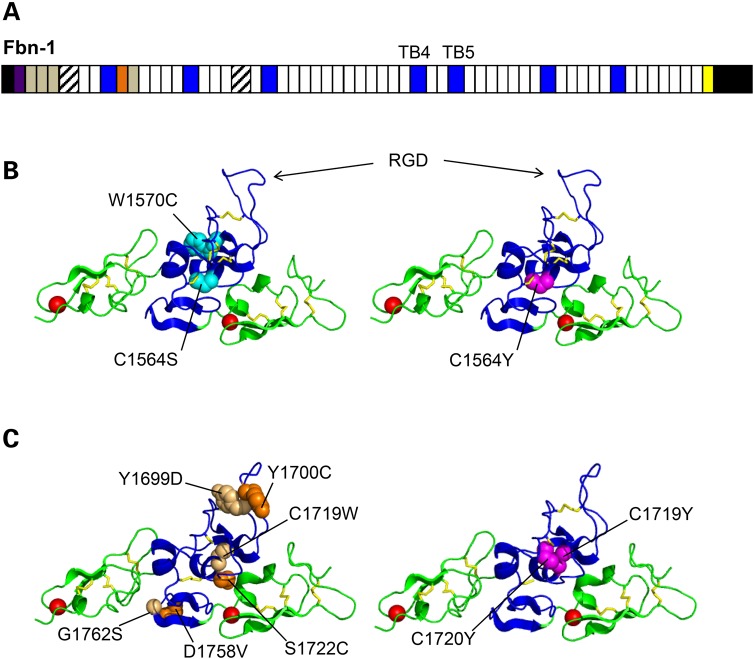
Fibrillin-1 domain organisation and mutation sites in domains TB4 and TB5. (**A**) The fibrillin-1 domain organisation is dominated by calcium-binding EGF-like domains (white) interspersed with transforming growth factor β-binding protein-like (TB; blue) and hybrid (diagonal stripes) domains. Other regions include the fibrillin unique N-terminal (FUN; purple) and non-calcium-binding EGF-like (grey) domains, a proline-rich region (orange) and a conserved 2Cys domain (yellow). N- and C-terminal propeptides (black) are processed by furin before microfibril assembly. (**B**) Structure of the cbEGF22-TB4-cbEGF23 fragment of fibrillin-1 (pdb 1UZJ) (6), with RGD integrin binding site indicated, showing the sites affected by SSS substitutions C1564S and W1570C (cyan spheres, left panel) and MFS substitution C1564Y (magenta spheres, right panel). In essence, residues important for stabilising TB domain fold are affected. (**C**) Homology model of the cbEGF24-TB5-cbEGF25 region of fibrillin-1, showing sites affected by geleophysic and acromicric dysplasias (light orange and dark orange spheres, respectively, left panel) and MFS substitutions (magenta spheres, right panel). All are predicted to affect the fold of the domain. The cbEGF24-TB5-cbEGF25 model was created with Modeller software using the cbEGF22-TB4-cbEGF23 structure as a template (10). Figures were rendered using PyMOL (Schrödinger, LLC) to show cbEGF domains (green), TB domains (blue), calcium ions (red) and disulphide bonds (yellow).

The structure of fibrillin-1 is dominated by 43 calcium-binding epidermal growth factor-like (cbEGF) domains interspersed with 7 transforming growth factor β-binding protein-like (TB) domains (Fig. 1A). TB domains are found only in the fibrillin/LTBP superfamily of proteins (11). Their structure is characterised by eight cysteine residues, disulphide bonded in a C1-C3, C2-C6, C4-C7, C5-C8 arrangement, with a small hydrophobic core that in most cases is formed by a conserved tryptophan (6,12,13). The majority of mutations affecting the gene encoding human fibrillin-1, *FBN1*, result in Marfan syndrome (MFS), a common (1:5000 incidence), autosomal dominant disorder of the fibrous connective tissue with highly variable clinical manifestations characterised by skeletal, ocular and cardiovascular defects. MFS-associated mutations affect all domains of fibrillin-1 and, until recently, few genotype–phenotype correlations have been established. An exception was the ‘neonatal’ region, spanning from domains cbEGF11-18, which is associated with a severe, early onset form of MFS (14).

Although the vast majority of *FBN1* mutations lead to MFS, in the past decade a series of disorders with phenotypes that contrast strongly with those observed in MFS have been linked to mutations affecting specific domains of fibrillin-1. Loeys *et al*. (15) identified three substitutions (C1564S, W1570C and C1577G) linked to stiff skin syndrome (SSS) and a further substitution (G1594N) linked to a hybrid syndrome featuring stiff skin with ectopia lentis. All of these result from mutations affecting domain TB4 of fibrillin-1 (Fig. 1B), which contains the only integrin-binding RGD motif of the molecule. In contrast to MFS, SSS is a congenital form of scleroderma characterised by the development of hard, thickened skin over most of the body, without the typical skeletal, ocular and cardiovascular features of MFS. In addition to SSS, several mutations affecting fibrillin-1 have been linked to another series of disorders, known collectively as the acromelic dysplasias (Fig. 1C). These syndromes, which include geleophysic dysplasia, acromicric dysplasia and Weill–Marchesani syndrome, are characterised by short stature, short extremities and joint stiffness, with skin thickening also present in some cases. At present, 16 missense mutations and an amino acid duplication, all affecting domain TB5, are known to result in either geleophysic dysplasia or acromicric dysplasia (16,17). Fibrillin-associated mutations causing the dominant form of Weill–Marchesani syndrome include two substitutions (R1596P and C1748R) and an eight amino acid deletion affecting domain TB5 (18–20), a substitution (G214S) in the first hybrid domain (21) and a deletion of exons 9–11 resulting in the loss of domains TB1 to EGF4 (19). Despite the phenotypic differences between SSS, the acromelic dysplasias and MFS, dysregulation of TGFβ is a prominent feature in all of these disorders (22).

Previous studies on the pathogenesis of MFS have examined the effects of mutations on aspects of the fibrillin-1 biosynthetic pathway including transcription (23,24), post-translational modification (25–27), susceptibility to degradation by proteases (28–31) and trafficking (25,32–34). Pulse chase studies of MFS patient ﬁbroblasts with missense mutations in FBN1 have shown three different cellular phenotypes of normal secretion, delayed secretion/ retention, and happloinsufficiency of fibrillin-1. In studies of a series of MFS-associated substitutions in cbEGF domains, both secreted and retained mutant proteins were described (32,33). Recently methods have become available to track the fate of specific fibrillin-1 mutant proteins into the assembling microfibrils (35,36) and have allowed the role of the C-terminal propeptide to be identified. The range of effects that have been observed to date suggest that all aspects of the microfibril biosynthetic pathway, from fibrillin-1 transcription to secretion, assembly and interactions at the cell surface, need to be investigated to understand disease pathogenesis. This will be especially important in disentangling the different pathogenic mechanisms leading to MFS, SSS and the acromelic dysplasias. Understanding these processes will provide new insights into how microfibrils influence integrin signalling and regulate growth factors such as TGFβ and the BMPs in the extracellular matrix.

In this study, we used a GFP-tagged version of full-length fibrillin-1 (36) and an established fibroblast secretion assay (32) to follow the secretion and microfibril incorporation of a series of fibrillin-1 TB domain mutants associated with MFS, SSS and acromelic dysplasias. We show that MFS-associated substitutions affecting domains TB4 and TB5 lead to a loss of fibrillin-1 from the cell culture medium whereas mutants associated with SSS and the acromelic dysplasias are secreted and detected in the extracellular media. The SSS and acromelic dysplasia-associated mutants were further shown to incorporate into the microfibril network produced by fibroblasts in culture. These data support a model in which SSS and the acromelic dysplasias result from changes in microfibril interactions post-assembly, rather than a loss of microfibrils as in MFS, and suggest important differences in the mechanisms leading to TGFβ dysregulation in these diseases.

## Results

### Fibrillin-1 domain TB4 SSS mutants are secreted and incorporate into microfibrils

**Figure 2.**
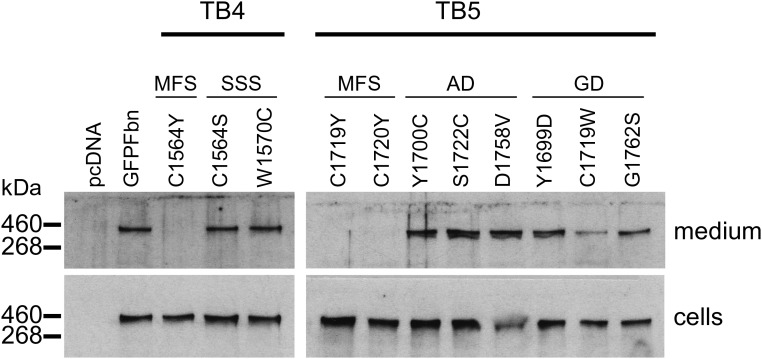
Secretion profiles of GFP-Fbn constructs with disease-associated substitutions. Mutations associated with MFS, SSS, geleophysic dysplasia (GD) or acromicric dysplasia (AD) in domains TB4 and TB5 were engineered into a GFP-tagged fibrillin-1 construct (36) and the resulting constructs used to transiently transfect HEK293T cells. After 3 days in culture, samples of the medium and cell lysates were analysed by western blotting following separation on a reducing 6% SDS-PAGE gel, using an anti-GFP antibody. SSS, GD and AD mutants were detected in the culture medium, in contrast to the MFS mutants. Empty vector (pcDNA) and the wild-type construct (GFP-Fbn) were used as negative and positive controls. Cell lysate samples showed that the lack of recombinant material in the media of the MFS mutants was not due to a loss of protein expression.

In a previous study using a fibrillin-1 mini-gene construct, we showed that the SSS-associated substitutions C1564S and W1570C in domain TB4 do not abrogate secretion of fibrillin-1 from fibroblasts (15). In this study, we compared the cell trafficking profiles of SSS substitutions C1564S and W1570C with a C1564Y substitution in domain TB4 associated with MFS (Fig. 1), in the context of a full-length GFP-tagged fibrillin-1 construct. Consistent with the fibrillin-1 mini-gene data, the GFP-Fbn constructs containing the SSS-associated substitutions C1564S and W1570C were detected in medium samples from cultures of transiently transfected HEK293T cells (Fig. 2). In contrast, the GFP-Fbn construct harbouring the MFS-associated substitution C1564Y was not detected in medium samples despite the recombinant protein being detected in cell fractions, suggesting either intracellular retention or a rapid turnover of the recombinant protein in the culture medium. Semi-quantitative RT–PCR analysis showed that the levels of recombinant transcript were similar in cells transfected with either the wild-type GFP-Fbn or C1564Y mutant (Supplementary Material, Fig. S1), indicating that the lack of recombinant protein observed in the medium in the C1564Y samples was not due to a lack of transcript.

**Figure 3.**
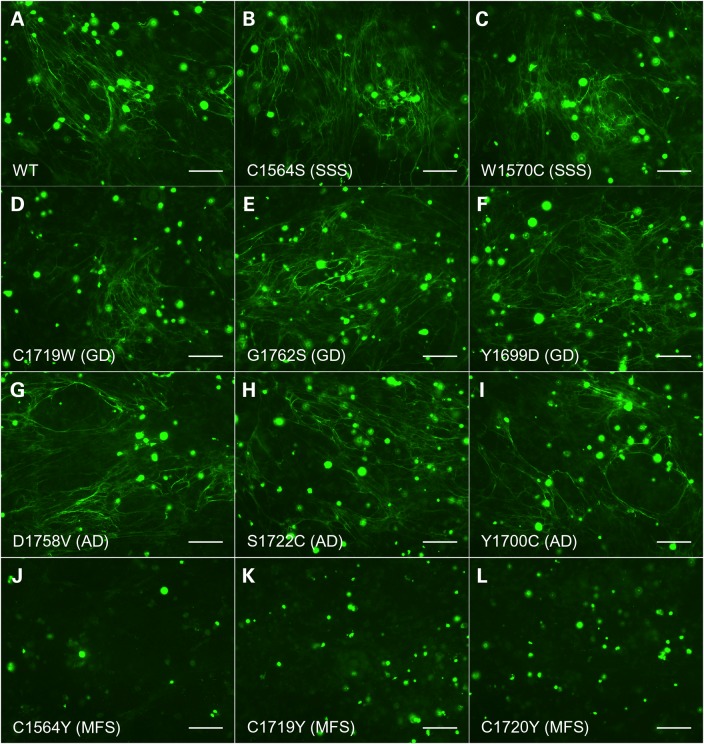
Microfibril incorporation of mutant GFP-Fbn constructs. FS2 fibroblasts were co-cultured for 5 days with HEK293T cells transiently transfected to express GFP-Fbn (WT; Panel **A**) or GFP-Fbn variants associated with SSS (Panels **B** and **C**), geleophysic dysplasia (GD; Panels **D**–**F**), acromicric dysplasia (AD; Panels **G**–**I**) or MFS (Panels **J**–**L**). Co-cultures were then fixed and stained using an anti-GFP antibody as described previously (36). SSS-associated mutants C1564S and W1570C, as well as the geleophysic dysplasia and acromicric dysplasia mutants, produced recombinant microfibril networks that were indistinguishable from the wild type. Co-cultures expressing the MFS mutants showed no recombinant microfibril staining, consistent with the lack of recombinant material observed in medium samples by western blotting (Fig. 2). Bar = 100 μm. Staining for endogenous fibrillin-1, expressed by the FS2 cells, is shown in Supplementary Material, Figure S2.

To assess the ability of fibrillin-1 molecules containing the SSS-associated substitutions to incorporate into microfibrils in culture, we used a microfibril incorporation assay (36) in which HEK293T cells transiently expressing wild-type GFP-Fbn (WT), GFP-FbnC1564S or GFP-FbnW1570C (Fig. 3, Panels A–C) were co-cultured with human dermal fibroblasts. As in the case of the wild-type recombinant protein, the GFP-Fbn variants containing the C1564S or W1570C substitutions were able to incorporate into the fibrillin-1 microfibril network. No GFP-tagged microfibrils were observed in cultures expressing the C1564Y construct (Fig. 3J), as expected by the lack of recombinant protein observed in medium samples from transiently transfected HEK293T cells. These data suggest that the C1564S- and W1570C-substituted fibrillin-1 mutants are not defective in their capacity to incorporate into microfibrils.

### Fibrillin-1 domain TB5 geleophysic dysplasia and acromicric dysplasia mutants are secreted and incorporate into microfibrils

When expressed in HEK293T cells, GFP-Fbn fusions with either geleophysic dysplasia or acromicric dysplasia-associated substitutions in TB5 were secreted into the culture medium (Fig. 2). In contrast, the MFS-associated substitutions C1719Y and C1720Y resulted in a lack of detectable protein in the media, suggesting either intracellular retention or an increased rate of turnover as in the case of the MFS-associated TB4 domain mutant, C1564Y. Semi-quantitative RT–PCR was carried out on RNA samples from cultures of HEK293T cells transfected with either the wild-type or MFS-associated GFP-Fbn constructs to ensure that the lack of secretion was not due to a lack of recombinant transcript (Supplementary Material, Fig. S1).

Using the GFP-Fbn microfibril binding assay, we found that the GFP-Fbn constructs containing substitutions associated with either geleophysic dysplasia (Fig. 3, Panels D–F) or acromicric dysplasia (Fig. 3, Panels G–I) showed incorporation into the fibroblast-derived microfibril network to a similar level as that seen for wild-type GFP-Fbn. As in the case of the TB4 domain C1564Y MFS-associated mutant, none of the TB5 domain MFS-associated GFP-Fbn constructs were detected in the microfibril network assembled by the FS2 fibroblasts in co-culture (Fig. 3, Panels K and L), consistent with the lack of secreted material observed by western blotting of medium samples. Staining of the cultures using an antibody against the fibrillin-1 proline-rich region showed that the microfibril network assembled by the fibroblasts in the co-cultures was not affected by the presence of the HEK293T cells expressing any of the disease-associated mutants (Supplementary Material, Fig. S2). These data indicate that the mutations associated with geleophysic dysplasia and acromicric dysplasia do not lead to an obvious change in the ability of fibrillin-1 monomers to assemble into microfibrils.

### Secretion profiles of fibrillin-1 domain TB4 MFS and SSS mutants from fibroblasts

**Figure 4.**
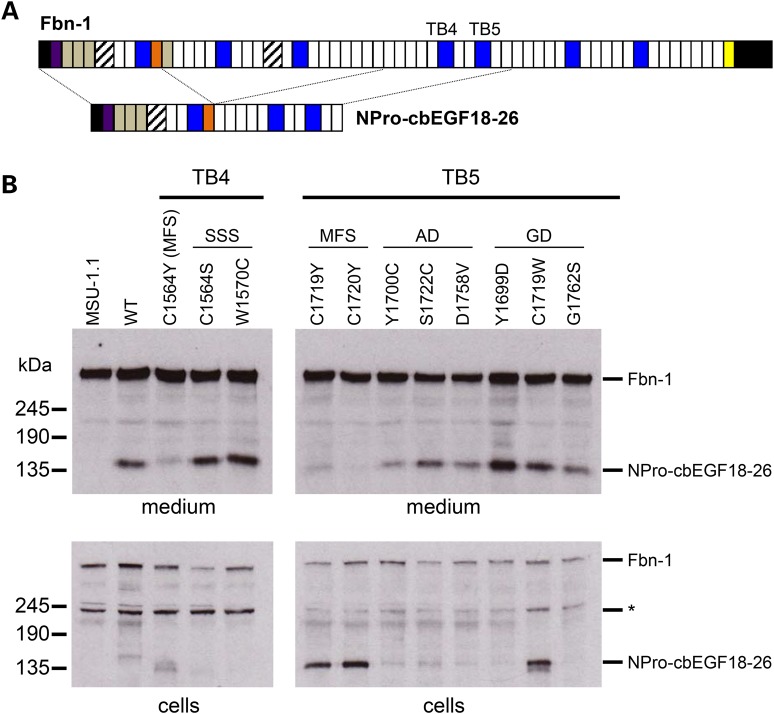
Construction of the NterPro-cbEGF18-26 fibrillin-1 fragments and fibroblast secretion assays. (**A**) NterPro-cbEGF18-26 is a fusion of the N-terminal region of fibrillin-1, up to the proline-rich domain (orange), with the cbEGF18-26 region, which encompasses domains TB4 and TB5. The smaller size of this construct allows it to be distinguished from full-length fibrillin-1, following reducing SDS-PAGE, in western blots of medium from stably transfected fibroblasts when developed with an antibody raised again the proline-rich domain. (**B**) Secretion profiles of TB4 and TB5 domain-mutant constructs associated with MFS, SSS, acromicric dysplasia (AD) or geleophysic dysplasia (GD). Medium and cell lysate samples from untransfected fibroblasts (MSU-1.1) and fibroblasts transfected with the wild-type control construct (WT) were run as controls, allowing the identification of the recombinant construct (fusion). The full-length fibrillin-1 band (Fbn-1) acts as an endogenously expressed loading control. In cell lysates, a band migrating at ∼230 kDa (*) is likely to be due to a degradation product of the endogenously expressed fibrillin-1.

To assess the effects of these substitutions on protein secretion from a microfibril-assembling cell line rather than HEK293T cells, which are epithelial in origin and do not assemble microfibrils, we used a previously described assay (32) in which pools of stably transfected clones of the human fibroblast cell line, MSU-1.1, are created to express a fibrillin-1 mini-gene (Fig. 4). MSU-1.1 fibroblasts are able to assemble extracellular microfibrils and contain all the cellular factors required for fibrillin-1 folding, processing, secretion and assembly (37). Pools of clones expressing the NPro-cbEGF18–26 fibrillin-1 mini-gene (Fig. 4A) were assayed to average out any variation in transgene expression levels caused by random genomic integration. Conditioned medium from pools of MSU-1.1 clones obtained after transfection with NPro-cbEGF18-26 wild-type, W1570C, C1564S or C1564Y constructs were analysed by western blotting using an antibody raised against the proline-rich region of fibrillin-1 (Fig. 4B). In medium samples from wild type, W1570C and C1564S pools, the recombinant NPro-cbEGF18-26 fragment, which migrates at ∼150 kD, was always detected in the medium. This band was absent in medium samples from untransfected MSU-1.1 cells, confirming that it is derived from the recombinant construct. In contrast, medium samples of pools expressing the C1564Y-substituted protein showed the presence of endogenous fibrillin-1 but greatly reduced the levels of the recombinant protein compared with the wild type and SSS forms. In addition, little C1564Y-substituted fusion was detected in the cell fraction, suggesting either a lack of expression or increased rate of turnover. Semi-quantitative RT–PCR analysis showed that the recombinant constructs were expressed at similar levels at the mRNA level (Supplementary Material, Fig. S3). The results suggest that the MFS-causing mutation C1564Y in domain TB4 results in a reduction in the amount of fibrillin-1 secreted by fibroblasts, consistent with the HEK293T data and in contrast to the SSS-causing mutants in domain TB4, which are secreted at levels comparable to the wild type.

### Secretion profiles of fibrillin-1 domain TB5 MFS and acromelic dysplasia mutants from fibroblasts

Although the majority of known missense mutations that affect fibrillin-1 domain TB5 result in the development of acromelic dysplasias (16), missense mutations associated with MFS have also been described (21,38,39). Using the fibroblast secretion assay and the NterPro-cbEGF18-26 construct, we compared the secretion profiles (Fig. 2) of two MFS-associated missense mutants (C1719Y and C1720Y) with the secretion profiles of mutants associated with geleophysic dysplasia (Y1699D, C1719W and G1762S) and acromicric dysplasia (Y1700C, S1722C and D1758V). The mutant fusion proteins with acromicric dysplasia-associated substitutions (AD) were mainly secreted into the medium, with little evidence of material being retained in the cells. In the case of geleophysic dysplasia-associated substitutions (GD), the Y1699D and G1762S variants were detected in the medium whereas the C1719W form was also found at increased levels in cell lysates, suggesting partial retention in this case. In contrast, only trace amounts of the MFS-associated substitutions C1719Y and C1720Y were detected in medium samples, with increased levels of the fusion protein retained within the cells. In all cases, semi-quantitative RT–PCR analysis was carried out using RNA extracted from all pools of clones to ensure that differences in secretion profiles were not due to the differences in the levels of transcript expression (Supplementary Material, Fig. S3). These data suggest that the MFS-associated TB5 domain substitutions studied here cause fibrillin-1 to be intracellularly retained whereas the acromelic dysplasia-associated TB5 domain mutants have secretion profiles similar to the wild type, except in the case of C1719W where there is also some intracellular retention detected.

## Discussion

We have investigated the effects on secretion and microfibril assembly of a series of substitutions in fibrillin-1 domains TB4 and TB5 that have been linked to syndromes with markedly different phenotypes. The mutants studied affected various parts of the TB domain structure, including an N-terminal loop adjacent to the C2-C6 disulphide bond (Y1699D and Y1700C), the hydrophobic core region (C1564S, C1564Y, W1570C, C1719Y, C1719W and S1722C) and the C-terminal β-sheet (D1758V and G1762S). While the substitutions outside of the hydrophobic core region were found to have little effect on protein secretion, there were mixed results from substitutions near the core of the domain. In the cases of residues C1564 and C1719, we observed opposing effects with different substitutions. In both cases, substitution of the cysteine residue with a tyrosine resulted in a loss of recombinant protein in the cell culture medium. The SSS-associated substitution, C1564S, was similar to wild type in terms of secretion and microfibril binding. It could be reasoned that this change, while causing the loss of a conserved disulphide, may lead to only localised misfolding because cysteine and serine are similar in size. The C1719W geleophysic dysplasia-associated substitution is of particular interest because its effects on secretion and microfibril assembly are different to those observed for the MFS-associated C1719Y substitution, even though in both cases cysteine is replaced with an aromatic residue. Apart from the difference in size between tryptophan and tyrosine side chains, an obvious difference is the presence of a hydroxyl group on tyrosine, which may lead to the formation of disruptive hydrogen bonds within the domain structure. Substitutions C1564Y and C1720Y, which are also associated with MFS, also result in a loss of protein in the medium, suggesting that tyrosine residues in these positions lead to more severe misfolding.

SSS-causing amino acid substitutions C1564S and W1570C affect structurally important residues of domain TB4 (Fig. 1B), which is the only domain within fibrillin-1 containing an integrin-binding RGD motif. The secretion of these mutant molecules is interesting considering that these substitutions, by definition, will result in misfolding. A tryptophan is found in the position corresponding to residue W1570 in almost all known TB domains of the fibrillins and LTBPs and is involved in forming the hydrophobic core of the domain (6,12,13). Residue C1564 is involved in forming one of the four conserved disulphide bonds that define the structure of the TB domain.

As in the case of the SSS mutants in domain TB4, the substitutions in domain TB5 associated with the acromelic dysplasias did not abolish secretion of either the GFP-Fbn construct from HEK293T cells or the NterPro-cbEGF18-26 construct from fibroblasts. Only the C1719W substitution, associated with geleophysic dysplasia, showed a degree of intracellular retention in the fibroblast system. Of the two acromelic dysplasias studied here, geleophysic dysplasia has the more severe phenotype with cardiovascular involvement (22). Apart from the partial intracellular retention we observed for the C1719W substitution, our data do not provide a clear explanation for the difference in disease phenotype. Some substitutions have been linked to both geleophysic dysplasia and acromicric dysplasia (16), suggesting that these diseases may form a spectrum of severity influenced by other modifying factors.

In previous studies on the secretion profiles of fibrillin-1 cbEGF domain mutants associated with MFS or the more severe neonatal form of MFS (32,33), no clear correlations were observed between disease severity and protein secretion. In the present study, the MFS-associated substitutions in domains TB4 and TB5 resulted in a loss of recombinant protein in the tissue culture medium, presumably due to intracellular retention. An increased susceptibility to extracellular protease digestion, however, cannot be excluded. Further analysis of other MFS-associated substitutions would be required to determine whether the apparent lack of secretion we observed is a common feature of mutations that affect TB domains.

The microfibril incorporation data presented suggest that the SSS-associated mutants are still able to assemble and incorporate into the matrix, although interactions with integrins may be disrupted owing to altered solvent accessibility or presentation of the RGD loop. This is consistent with previous work showing that the replacement of the RGD integrin-binding motif with an RGA sequence does not disrupt microfibril assembly (35). Our data suggest that cell–matrix interactions between integrins and microfibrils, downstream of microfibril assembly, must be altered in SSS. This would be consistent with the data from a mouse model of SSS obtained by Gerber *et al*. (40) who showed that dermal fibrosis in mutant animals could be reversed by integrin-modulating therapies. SSS substitutions C1564S and W1570C were previously shown to induce a loss of attachment and spreading of fibroblasts plated onto fibrillin-1 fragments containing domain TB4 (cbEGF22-TB4-cbEGF23), indicating an impaired interaction of these fragments via the RGD motif with the integrin αvβ3 (15). This suggests that the structural consequences of the C1564S and W1570C mutations extend to the RGD loop of domain TB4 either through short-range effects limited to the TB domain or by disruption of the extensive hydrophobic interfaces that occur between domain TB4 and the adjacent cbEGF domains (6,41). The interaction with integrin αvβ6 was still viable however, suggesting that the RGD motif could still bind to a smaller repertoire of integrins.

Domain TB5 has been implicated as a region on fibrillin-1 involved in binding to heparan sulphate proteoglycans (HSPGs) (42). A 24-bp in-frame deletion in domain TB5 linked to Weill–Marchesani syndrome and missense mutants linked to the geleophysic and acromicric dysplasias have been reported to alter the interactions of this region of fibrillin-1 with heparan sulphate (43). Although there is substantial evidence for the role of cell-surface HSPGs in the assembly of fibrillin microfibrils (42,44–46), a specific interaction between a cell-surface HSPG and fibrillin-1 is still to be demonstrated biochemically. In marked contrast to the TB5 domain MFS mutants, all of the acromelic dysplasia-associated mutants we investigated were able to associate with fibroblast-derived microfibrils in the GFP-Fbn co-culture assay. This suggests that, as in the case of the SSS mutants, the acromelic dysplasia mutants may be acting at the level of cell–matrix interactions rather than disrupting microfibril assembly and that heparan sulphate interactions involving this region of fibrillin-1 are not critical for assembly. Bax *et al*. (47) showed that heparan sulphate-binding modulates integrin binding to the TB4 domain of fibrillin-1. Integrin and cell-surface HSPGs could interact in concert to regulate signalling on binding to microfibrils. Syndecans, which are a group of cell-surface proteoglycans, are able to modulate integrin signalling at focal adhesions (48) and are a potential binding partner for the fibrillin-1 TB5 domain. Disruption of either the integrin or HSPG interaction may alter cellular responses, leading to changes in the expression levels of other matrix components or growth factors, and ultimately lead to the development of either SSS or an acromelic dysplasia.

**Figure 5.**
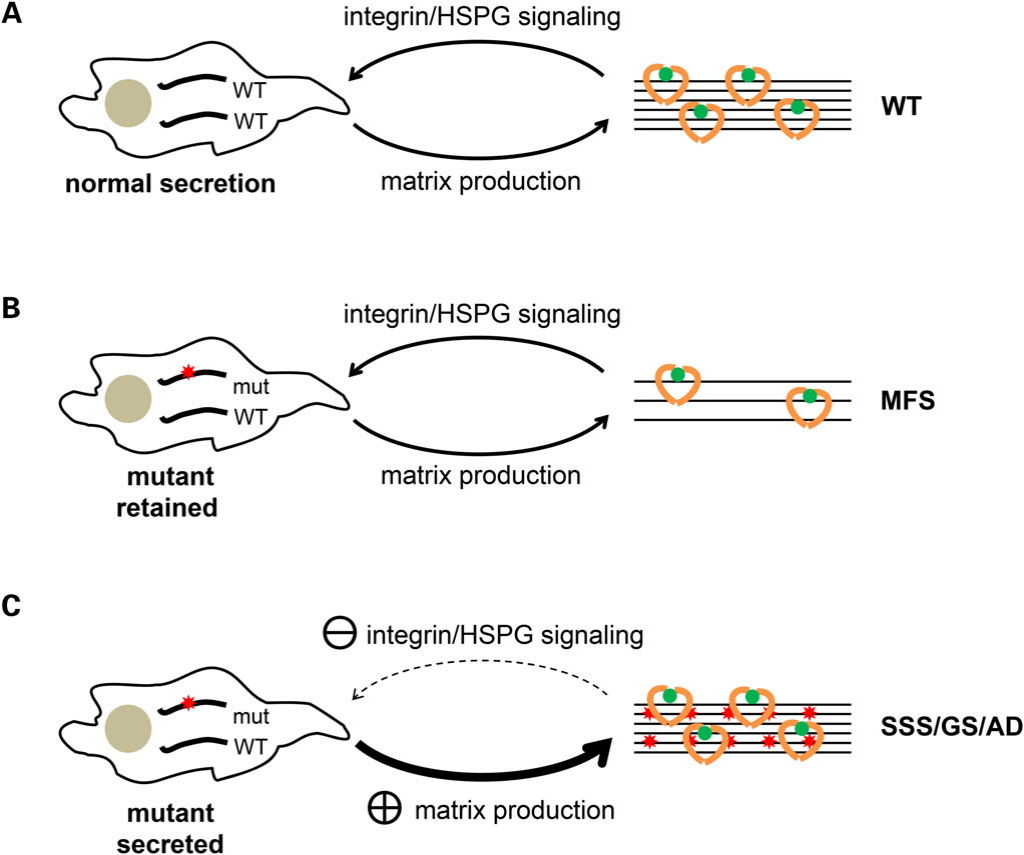
Model of the mechanisms leading to different dominantly inherited fibrillinopathies associated with fibrillin-1 domains TB4 and TB5. (A) Wild-type fibrillin-1 is secreted from cells and assembled to produce a microfibril network that influences cell signalling through integrins and cell-surface HSPGs. Normal matrix deposition of growth factors (green and yellow), and growth factor activation, is mediated by interactions between cells and microfibrils (curved arrows). (B) When one of the alleles carries a pathogenic mutation that results in intracellular retention of the mutant protein, fewer functional microfibrils are produced in all tissues. Altered tissue dynamics, and possibly a reduction in growth factor binding sites in the matrix, lead to a general increase in growth factor activation and the development of MFS, but with microfibrils maintaining normal interactions with integrins and cell-surface HSPGs. (C) Alternatively, the mutant proteins could be secreted and assembled into structurally normal microfibrils, with defective interactions between microfibrils and integrins and/or cell-surface HSPGs being affected by altered binding site presentation. The resulting alterations in signalling lead to a secondary upregulation in the production of extracellular matrix. In these cases, the resulting phenotype is limited to tissues expressing the cell-surface proteins most sensitive to the altered binding sites on the microfibril, as seen in SSS and the acromelic dysplasias.

The role of TGFβ in the pathogenesis of MFS was initially demonstrated by Neptune *et al*. (49) in a mouse model, and current data from both patient samples and animal models suggest that the fibrosis observed in SSS and the acromelic dysplasias is also due to TGFβ dysregulation (15,50,51). The mechanisms leading to TGFβ dysregulation, and how they result in the different phenotypes observed in these syndromes, remains unclear. Our data show that MFS-associated substitutions in domains TB4 and TB5 reduce the concentration of fibrillin-1 found in the medium of cultured cells, with a corresponding lack of mutant protein being incorporated into microfibrils. This is consistent with a dominant mechanism in which a functional haploinsufficiency of fibrillin-1 or loss of microfibrils is at the heart of the pathogenesis of MFS, as shown by mouse models and human deletion mutants (52,53). Based on the ability of the non-MFS TB domain mutants presented here to secrete and be incorporated into microfibrils, we propose that the phenotypes of SSS and the acromelic dysplasias are not caused by a primary defect in microfibril assembly. Instead, these mutants are more likely to alter the cell–matrix interactions involving microfibrils (Fig. 5) leading to secondary effects on the extracellular matrix, such as fibrosis. The types of alterations would depend on the repertoire of cell-surface proteins in particular tissues and would explain the more limited range of tissues affected in SSS and the acromelic dysplasias compared with MFS. We therefore propose that the aberrant TGFβ signalling observed in each of the fibrillin-1-associated diseases is due to different causes. In MFS, we suggest that it is due to a loss of structural integrity in the fibrillin matrix, which affects TGFβ activation through a change in the mechanical properties of tissues. In contrast, the primary defect in SSS and the acromelic dysplasias is more likely to be defective cell-surface interactions with microfibrils rather than a defect in microfibril assembly or stability. The TGFβ dysregulation, fibrosis and dermal accumulation of microfibril aggregates observed in SSS and the acromelic dysplasias would then be a secondary response to an altered signalling program initiated by changes in the surface interactions with microfibrils. Further molecular and cellular studies will be required to investigate differences in cellular signalling in the different disorders.

## Materials and Methods

### Plasmid construction, mutagenesis and transfection

Construction of the plasmid pcDNA-GFP-Fbn has been described previously (36). Plasmids for the expression of the NterPro-cbEGF18-26 constructs were created by amplifying the DNA encoding residues T1321 to N1848 as a *Sal*I fragment from a fibrillin-1 cDNA clone and inserting this into the *Xho*I site of the plasmid pKG52(polyA) (32), which encodes the N-terminal region of fibrillin-1 up to the proline-rich domain (residues 1–446) under the control of a thymidine kinase promoter. Mutant constructs were created using by an overlapping PCR method (41) or the QuikChange Lightning mutagenesis kit (Agilent).

Transfections were carried out using Lipofectamine 2000 (Life Technologies) according to the manufacturer's instructions. Pools of stable, puromycin-resistant cell lines expressing NterPro-cbEGF18-26 constructs were created using MSU-1.1 fibroblasts (typically >80 clones per pool) as described previously (32,36,54). Pooling of clones was used to average out the effects of variations in expression levels between individual clones resulting from random genomic integration and to overcome the low transient transfection efficiency of fibroblasts. Transfections of HEK293T cells with GFP-Fbn constructs were carried out in six-well plates as described previously (36).

### Microfibril incorporation assay

Microfibril incorporation assays were carried out as described previously (36). Briefly, HEK293T cells grown in six-well plates were transfected with GFP-Fbn constructs using Lipofectamine 2000. Twenty-four hours after transfection, cells were trypsinised and counted. Co-cultures were established containing 7.5 × 10^4^ FS2 dermal fibroblasts (15) and 7.5 × 10^4^ transfected HEK293T cells per well of an eight-well Lab Tek II chambered slide, in 400 μl Dulbecco's modified Eagle medium supplemented with 2 mm glutamine, 50 U/ml penicillin, 50 μg/ml streptomycin and 10% (v/v) foetal bovine serum (complete DMEM). After 5 days in culture, cells were fixed with 4% (w/v) paraformaldehyde in phosphate-buffered saline and stained using a rabbit polyclonal antibody raised against the fibrillin-1 proline-rich region (32) and chicken polyclonal anti-GFP (Abcam; ab13970) without permeabilisation. Goat anti-chicken Alexa488 and goat anti-rabbit Alexa568 (Invitrogen) were used for detection. Images were collected using a Zeiss Axioplan 2 microscope with AxioVision Rel. 4.8 software.

### Immunoblotting

Cells remaining from the HEK293T transfections were transferred to 25-cm^2^ tissue culture flasks (Greiner) and grown for a further 3 days in complete DMEM to produce cell and medium samples for analysis by western blotting. Conditioned media and cell samples were analysed by immunoblotting as described previously (32,33), using a chicken anti-GFP antibody followed by a goat anti-chicken HRP conjugate and enhanced chemiluminescent detection (Amersham).

## Supplementary Material

Supplementary Material is available at *HMG* online.

*Conflict of Interest statement*. None declared.

## Funding

This work was supported by Arthritis Research UK grant 19810. Funding to pay the Open Access publication charges for this article was provided by The Charity Open Access Fund.

## Supplementary Material

Supplementary Data
