# Supplementary material for: A microfibril assembly assay identifies different mechanisms of dominance underlying Marfan syndrome, stiff skin syndrome and acromelic dysplasias
Source: Hum Mol Genet. 2015 May 15;24(15):4454–63. doi: 10.1093/hmg/ddv181 (PMC4492404; doi:10.1093/hmg/ddv181)
Supplement: Supplementary Data [file supp_24_15_4454__index.html]

A microfibril assembly assay identifies different mechanisms of dominance underlying Marfan syndrome, stiff skin syndrome and acromelic dysplasias — A microfibril assembly assay identifies different mechanisms of dominance underlying Marfan syndrome, stiff skin syndrome and acromelic dysplasias — Supplementary Data 

# A microfibril assembly assay identifies different mechanisms of dominance underlying Marfan syndrome, stiff skin syndrome and acromelic dysplasias

## Supplementary Data

Supplementary Data

- Supplementary Data - pptx file
