# Supplementary material for: A microfibril assembly assay identifies different mechanisms of dominance underlying Marfan syndrome, stiff skin syndrome and acromelic dysplasias
Source: Hum Mol Genet. 2015 May 15;24(15):4454–63. doi: 10.1093/hmg/ddv181 (PMC4492404; doi:10.1093/hmg/ddv181)
Supplement: Supplementary Data [file supp_ddv181_ddv181supp.pptx]

## Slide 1
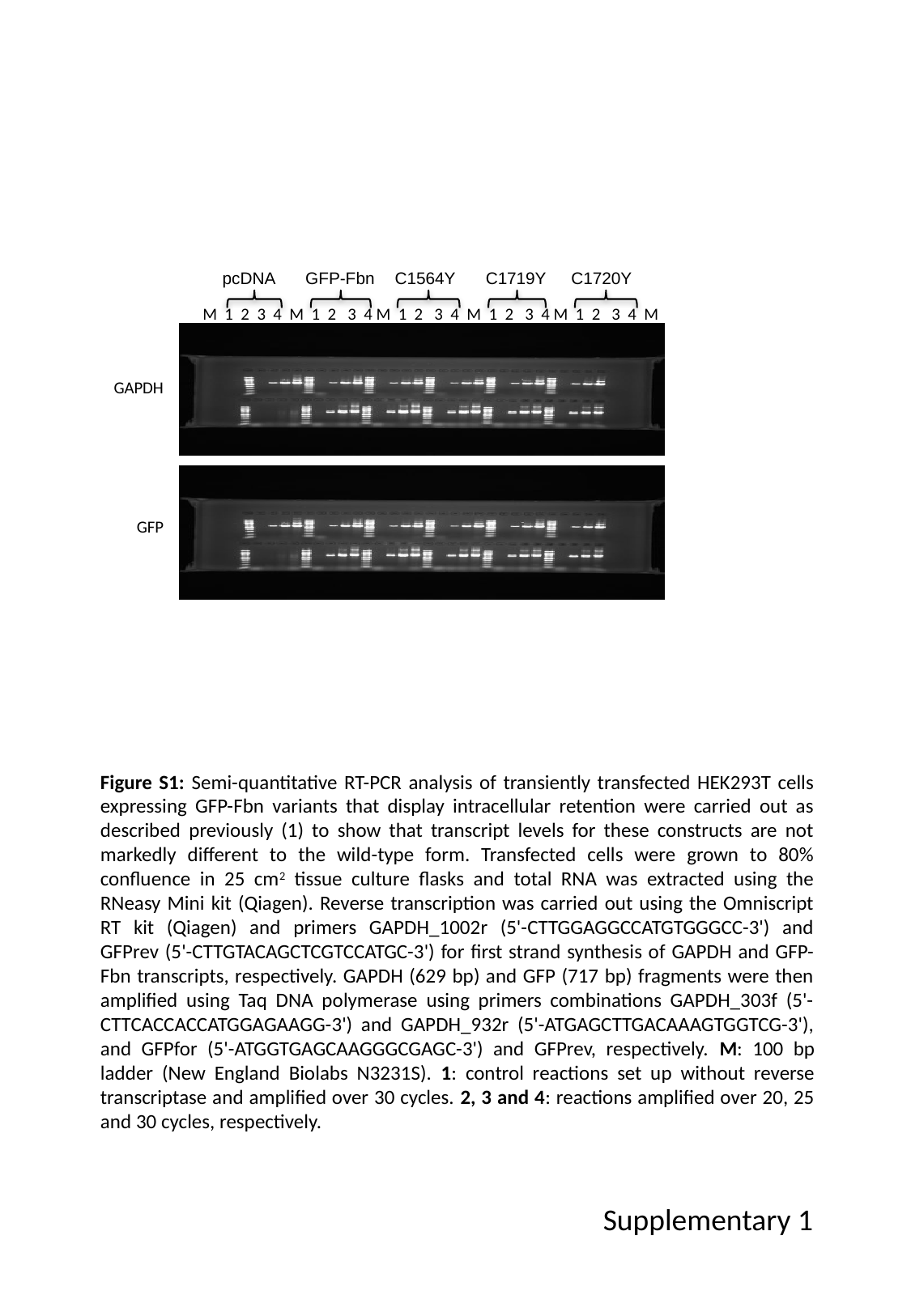

pcDNA
GFP-Fbn
C1564Y
C1719Y
C1720Y
M 1 2 3 4 M 1 2 3 4 M 1 2 3 4 M 1 2 3 4 M 1 2 3 4 M
GAPDH
GFP
Figure S1: Semi-quantitative RT-PCR analysis of transiently transfected HEK293T cells expressing GFP-Fbn variants that display intracellular retention were carried out as described previously (1) to show that transcript levels for these constructs are not markedly different to the wild-type form. Transfected cells were grown to 80% confluence in 25 cm2 tissue culture flasks and total RNA was extracted using the RNeasy Mini kit (Qiagen). Reverse transcription was carried out using the Omniscript RT kit (Qiagen) and primers GAPDH_1002r (5'-CTTGGAGGCCATGTGGGCC-3') and GFPrev (5'-CTTGTACAGCTCGTCCATGC-3') for first strand synthesis of GAPDH and GFP-Fbn transcripts, respectively. GAPDH (629 bp) and GFP (717 bp) fragments were then amplified using Taq DNA polymerase using primers combinations GAPDH_303f (5'-CTTCACCACCATGGAGAAGG-3') and GAPDH_932r (5'-ATGAGCTTGACAAAGTGGTCG-3'), and GFPfor (5'-ATGGTGAGCAAGGGCGAGC-3') and GFPrev, respectively. M: 100 bp ladder (New England Biolabs N3231S). 1: control reactions set up without reverse transcriptase and amplified over 30 cycles. 2, 3 and 4: reactions amplified over 20, 25 and 30 cycles, respectively.
Supplementary 1

## Slide 2
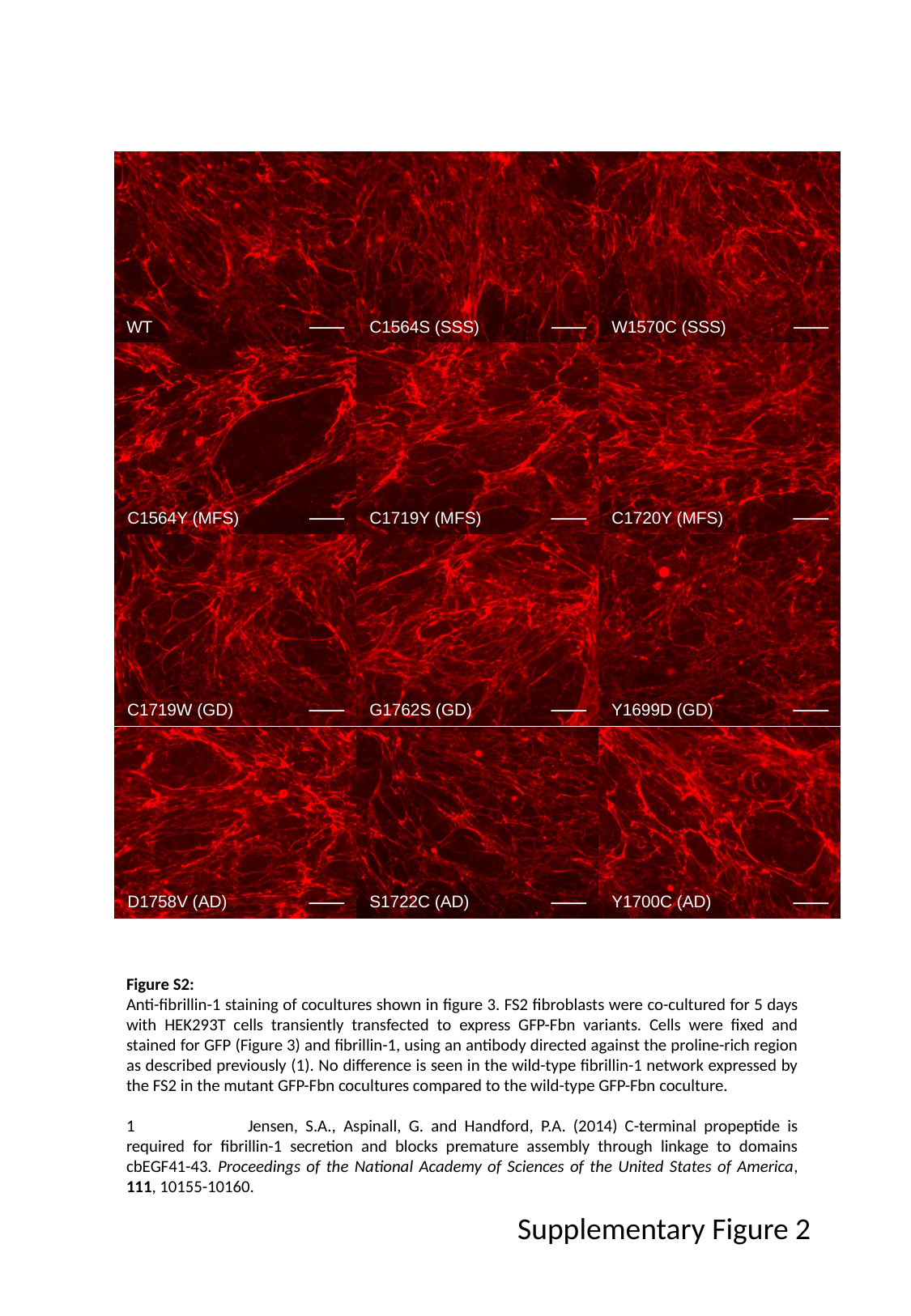

WT
C1564S (SSS)
W1570C (SSS)
C1564Y (MFS)
C1719Y (MFS)
C1720Y (MFS)
C1719W (GD)
Y1699D (GD)
G1762S (GD)
D1758V (AD)
S1722C (AD)
Y1700C (AD)
Figure S2:
Anti-fibrillin-1 staining of cocultures shown in figure 3. FS2 fibroblasts were co-cultured for 5 days with HEK293T cells transiently transfected to express GFP-Fbn variants. Cells were fixed and stained for GFP (Figure 3) and fibrillin-1, using an antibody directed against the proline-rich region as described previously (1). No difference is seen in the wild-type fibrillin-1 network expressed by the FS2 in the mutant GFP-Fbn cocultures compared to the wild-type GFP-Fbn coculture.
1	Jensen, S.A., Aspinall, G. and Handford, P.A. (2014) C-terminal propeptide is required for fibrillin-1 secretion and blocks premature assembly through linkage to domains cbEGF41-43. Proceedings of the National Academy of Sciences of the United States of America, 111, 10155-10160.
Supplementary Figure 2

## Slide 3
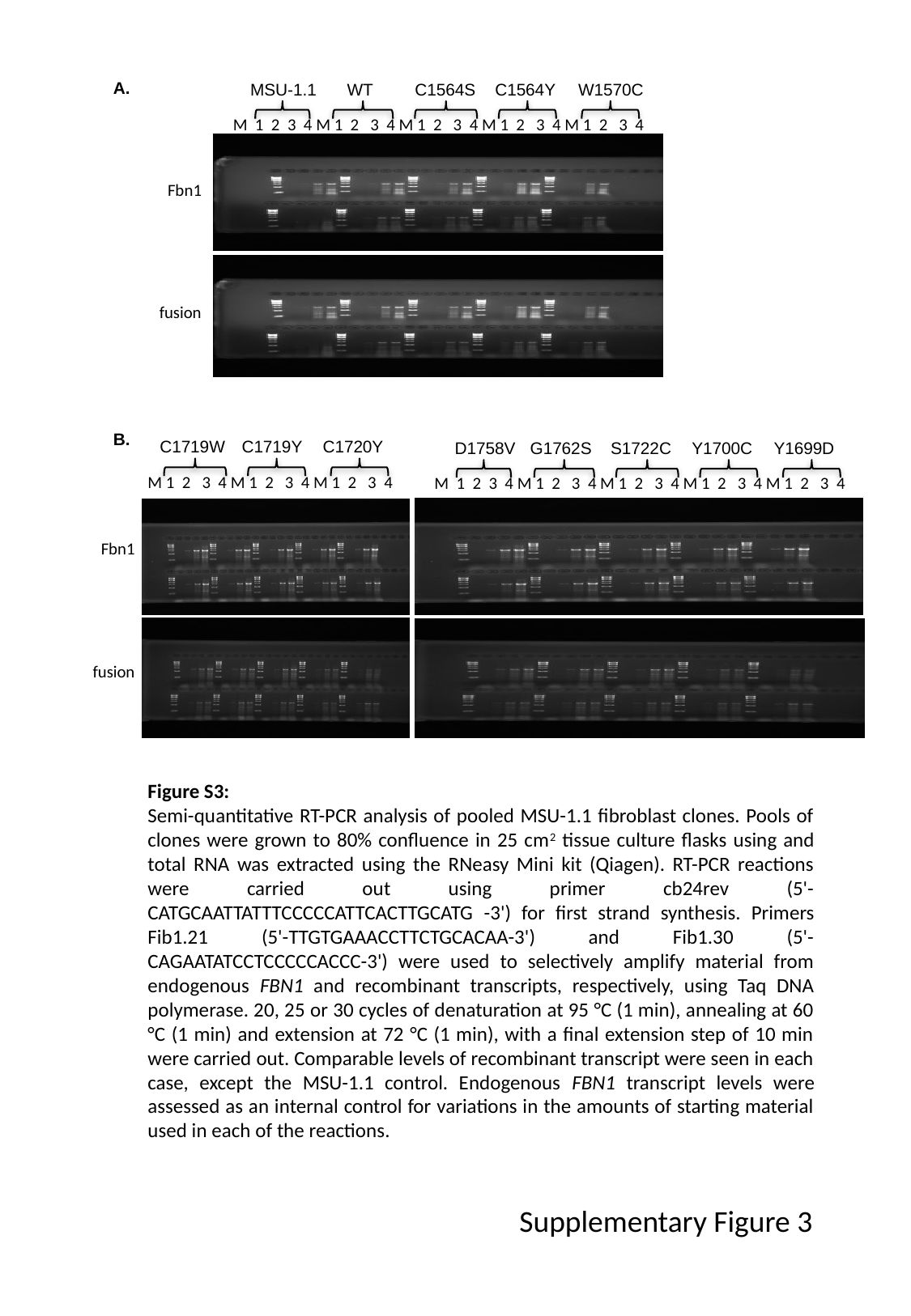

A.
MSU-1.1
WT
C1564S
C1564Y
W1570C
M 1 2 3 4 M 1 2 3 4 M 1 2 3 4 M 1 2 3 4 M 1 2 3 4
Fbn1
fusion
B.
C1719W
C1719Y
C1720Y
D1758V
G1762S
S1722C
Y1700C
Y1699D
M 1 2 3 4 M 1 2 3 4 M 1 2 3 4 M 1 2 3 4 M 1 2 3 4
M 1 2 3 4 M 1 2 3 4 M 1 2 3 4
Fbn1
fusion
Figure S3:
Semi-quantitative RT-PCR analysis of pooled MSU-1.1 fibroblast clones. Pools of clones were grown to 80% confluence in 25 cm2 tissue culture flasks using and total RNA was extracted using the RNeasy Mini kit (Qiagen). RT-PCR reactions were carried out using primer cb24rev (5'- CATGCAATTATTTCCCCCATTCACTTGCATG -3') for first strand synthesis. Primers Fib1.21 (5'-TTGTGAAACCTTCTGCACAA-3') and Fib1.30 (5'-CAGAATATCCTCCCCCACCC-3') were used to selectively amplify material from endogenous FBN1 and recombinant transcripts, respectively, using Taq DNA polymerase. 20, 25 or 30 cycles of denaturation at 95 °C (1 min), annealing at 60 °C (1 min) and extension at 72 °C (1 min), with a final extension step of 10 min were carried out. Comparable levels of recombinant transcript were seen in each case, except the MSU-1.1 control. Endogenous FBN1 transcript levels were assessed as an internal control for variations in the amounts of starting material used in each of the reactions.
Supplementary Figure 3
